# Basal ganglia echogenicity in tauopathies

**DOI:** 10.1007/s00702-014-1310-3

**Published:** 2014-09-10

**Authors:** Krzysztof Sadowski, Małgorzata Serafin-Król, Karol Szlachta, Andrzej Friedman

**Affiliations:** 1 Department of Neurology, Health Science Faculty, Warsaw Medical University, Kondratowicza 8, 03-242 Warsaw, Poland; 2 Department of Diagnostic Imaging, Second Medical Faculty, Warsaw Medical University, Warsaw, Poland; 3 Faculty of Physics, Warsaw University of Technology, Warsaw, Poland

**Keywords:** Transcranial sonography, Atypical Parkinsonism, Substantia nigra echogenicity

## Abstract

Accumulating data confirm the usefulness of transcranial sonography (TCS) in the diagnosis of Parkinson’s disease. The relevance of basal ganglia abnormalities depicted by TCS in atypical parkinsonian syndromes still needs further assessment. In the present study, 20 patients with progressive supranuclear palsy (PSP) and 13 patients with corticobasal syndrome (CBS) were studied with the use of transcranial sonography. Echogenicity of the substantia nigra (SN) and lenticular nucleus (LN) were assessed. 0/20 patients with PSP and 8/12 (66.6 %) patients with CBS were characterized with SN hyperechogenicity. LN hyperechogenicity was observed in 9/20 patients diagnosed with PSP and 0/11 of CBS patients. The combination of SN isoechogenicity and LN hyperechogenicity reached 100 % sensitivity and positive predictive value for the diagnosis of PSP. The results of this study point out that CBS has to be taken into consideration when SN hyperechogenicity is depicted in a patient with parkinsonian syndrome. Normal echogenicity of the SN coexisting with LN hyperechogenicity practically excludes CBS.

## Introduction

In the wake of the increasing need for reliable preclinical diagnosis of Parkinson`s disease (PD), numerous diagnostic tools are under investigation. Neuroimaging studies are among the most promising. SN hyperechogenicity depicted by TCS is a sensitive marker for PD (Becker et al. 1995). Consecutive studies (Berg et al. 1999) confirmed the reliability of TCS as a supporting diagnostic tool in PD patients. Due to a more limited data the appearance of SN hyperechogenicity in atypical Parkinsonism (APS) is still a matter of controversy. According to available data (Walter et al. 2003; Behnke et al. 2005) 6–25 % of patients with multiple system atrophy (MSA) are characterized by SN hyperechogenicity, whereas in patients diagnosed with progressive supranuclear palsy (PSP) this echo feature is observed in 8–47 %. To date there is only one published study (Walter et al. 2004) describing TCS findings in merely 8 patients diagnosed with corticobasal syndrome (CBS). Walter et al. reported high incidence (ca. 90 %) of bilaterally marked SN hyperechogenicity in CBS patients. Due to discrepant data SN hyperechogenicity might be of diverse specificity in the differential diagnosis of parkinsonian syndromes when comparing different populations. Hyperechogenicity of the lenticular nucleus (LN) is a frequent (72–88 % in available literature) and valuable supporting finding (Walter et al. 2003, 2004; Behnke et al. 2005) in APS but lacks specificity for particular disease entities. Due to clinical overlap and limited TCS data patients with tauopathies constitute a group of special interest. In the present study, SN and LN echogenicity assessment was applied to patients with two types of tauopathies—PSP and CBS—to evaluate the clinical usefulness of TCS as a supporting tool in the diagnosis of these conditions.

## Patients and methods

A total of 34 consecutive patients fulfilling diagnostic criteria for clinically probable PSP (*N* = 20) and CBS (*N* = 14) were recruited from in- and outpatient clinic (see Table 1 for details). All patients gave written consent for the study. The study obtained IRB approval. TCS was performed using a phased-array ultrasound system with a 1–4 MHz transducer (MyLab 70XVision, Esaote, Italy). SN and LN echogenicity were assessed according to available guidelines (Walter et al. 2007). The examiner was blinded to final clinical diagnosis, but not separated from the patient during TCS examination. The examination was performed bilaterally through temporal acoustic window with penetration depth of 16 cm and dynamic range of 40–45 dB. At the midbrain plane butterfly-shaped midbrain was depicted. The echogenic area of the ipsilateral SN was manually encircled and automatically measured. As in previous studies, the cut-off value of ≥0.20 cm^2^ on at least one side defined SN hyperechogenicity and ≥0.25 cm^2^- marked hyperechogenicity. By tilting the probe 10° in the upward direction the thalamus plane was visualized. At this level the contralateral lenticular nucleus was assessed qualitatively as iso- or hyperechogenic. The third ventricle width was not evaluated. Due to the authors opinion this measure, however undoubtedly valuable, might be as well obtained with the use of standard MRI imaging whereas basal ganglia echogenicity assessment brings new and unique diagnostic data unavailable with the use of other imaging modalities. Obtained images were assessed by two raters (M.S.-K. and K.S.) to increase the final reliability of measures; final picture analysis for each patient was performed jointly. The Chi-square test was employed to compare the results in both groups.

**Table 1:** Demographic and clinical data of patients

	PSP	CBS
Number	20	14
Sex M/F	12/8	7/7
Age (years, ±SD)	60 ± 5.86	64 ± 9.48
Disease duration (years, ±SD)	3.9 ± 2.3	3.5 ± 2.19
UPDRS III score (mean, ±SD)	42 ± 3	44 ± 5
Dementia	11/20	7/14
Alien limb syndrome	0/20	6/14

## Results

**Fig. 1:**
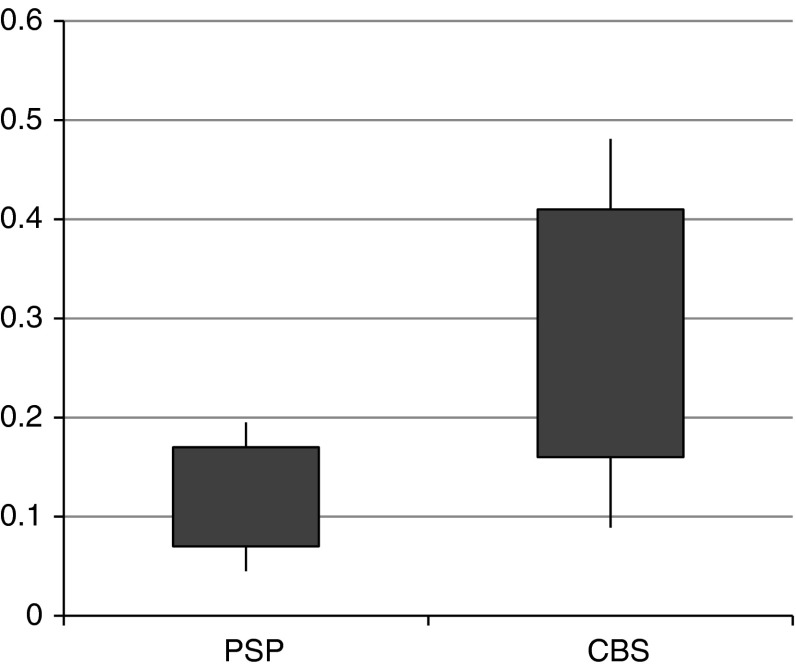
Comparison of the area of echogenicity in the SN (cm^2^) in PSP and CBS patients (*p* < 0.05)

All patients with PSP and 12 patients with CBS had sufficient acoustic temporal window. No significant difference in demographic data was observed (*p* > 0.05). SN hyperechogenicity was observed in 8 out of 12 (66.6 %) patients diagnosed with CBS. In all these cases the criteria for marked hyperechogenicity (≥0.25 cm^2^) were fulfilled. In all cases observed hyperechogenicity was unilateral and located contralaterally to the more affected side. None of PSP patients fulfilled the criteria for SN hyperechogenicity. The median size of 0.3 cm^2^ for CBS and 0.1 cm^2^ for PSP was observed (Fig. 1; *p* < 0.05). The larger echogenic side was taken into consideration in calculations. Assuming that the isoechogenic SN is characteristic for PSP the positive predictive value for PSP diagnosis reached 83.33 %, specificity 66.66 % and sensitivity 100 %. LN hyperechogenicity was depicted in 9 out of 20 patients with PSP and was not observed in patients diagnosed with CBS (*p* < 0.05). The combination of SN isoechogenicity and LN hyperechogenicity reached 100 % sensitivity and positive predictive value as a characteristic feature of CBS patients.

## Discussion

The results of this study confirm the relatively high occurrence of SN hyperechogenicity in patients diagnosed with CBS distinguishing them from other patients with atypical parkinsonian syndromes. Walter et al. (2004) reported that 7/8 (88 %) patients with CBS had marked bilateral SN hyperechogenicity (in comparison to 66.6 % and asymmetric in this study). In both studies none of the PSP patients showed this feature. Lenticular nucleus hyperechogenicity in this study was observed only in patients with PSP, whereas Walter et al. noted this echo feature in both groups with comparative frequency. The dynamic range setting at the level of 40–45 dB used in this study might result in higher contrast of TCS images enabling clear interpretation, but at the cost of slightly lower incidence of SN hyperechogenicity in comparison to Walter et al. (45–50 dB used). Patients with CBS appear to have distinctive features in TCS examination. The fact that SN hyperechogenicity is a frequent finding in CBS patients has to be taken seriously into consideration, especially in the context of the specificity of this finding in PD patients. In a patient with newly diagnosed parkinsonian syndrome and SN hyperechogenicity, CBS has to be taken into account. Marked SN hyperechogenicity, when considered in a proper clinical context, might support the diagnosis of corticobasal syndrome. Due to frequent overlap of symptomatology in PSP and CBS transcranial sonography findings might be also of value in more advanced cases. The pathophysiology of observed changes is still at the centre of debate. SN hyperechogenicity in PD patients is considered to be a result of iron storage dysregulation (Zecca et al. 2005). The absolute increase of iron within a substantia nigra of PD patients raises concerns (Wypijewska et al. 2010). Thus, it can be argued that the progressive loss of dopaminergic neurons with simultaneous microglial activation might explain the change in tissue sonographic features (Berg et al. 2010; Sadowski et al. 2012). This mechanism might be universal for diverse neurodegenerative disorders with substantia nigra involvement. The observation of significant SN hyperechogenicity in CBS cases might be, at least theoretically related to the very intense tau pathology in corticobasal degeneration. One may also assume that the asymmetry of the pathology of the CBS patients creates an artifact that looks like SN hyperechogenicity. An analogous argument can be made for the lentiform nucleus isoechogenicity. Interpretation of LN hyperechogenicity observed in atypical parkinsonian syndromes meets with difficulties. There are few research groups applying this part of TCS examinations. A more subjective way of LN echogenicity assessment (qualitative analysis) is another source of potential difficulties. Thus, results are more ambiguous in comparison to SN echogenicity studies. Most probably LN hyperechogenicity is characteristic for atypical parkinsonian syndromes. No studies explaining the pathogenesis of LN hyperechogenicity in atypical parkinsonian syndromes were conducted so far. Due to high incidence of clinical–pathological mismatch in CBS patients (only 50 % of patients clinically presenting as corticobasal syndrome have corticobasal degeneration) neuropathological studies would be of key importance. Would the neurodegenerative process within the SN in PSP and CBS differ in a way affecting the sonographic imaging, will probably be explained in consecutive studies. Short period of observation and lack of pathological confirmation are obvious drawbacks of this study. Thus, the results are useful as justification for further in-depth studies. On the other hand, obtained results might be important for everyday clinical practice and broaden our knowledge of practical use of transcranial sonography in movement disorders.

## References

[CR1] Becker G, Seufert J, Bogdahn U, Reichmann H, Reiners K (1995). Degeneration of the substantia nigra in chronic Parkinson`s disease visualized by transcranial color-coded real-time sonography. Neurology.

[CR2] Behnke S, Berg D, Naumann M, Becker G (2005). Differentiation of Parkinson’s disease and atypical parkinsonian syndromes by transcranial ultrasound. JNNP.

[CR3] Berg D, Becker G, Zeiler B, Tucha O, Hofmann E, Preier M (1999). Vulnerability of the nigrostriatal system as detected by transcranial ultrasound. Neurology.

[CR4] Berg D, Godau J, Riederer P, Gerlach M, Arzberger T (2010). Microglia activation is related to substantia nigra echogenicity. J Neural Transm.

[CR5] Sadowski K, Szlachta K, Serafin-Król M, Gałązka-Friedman J, Friedman A (2012). Brain tissue echogenicity- implications for substantia nigra studies in parkinsonian patients. J Neural Transm.

[CR6] Walter U, Niehaus L, Probst T, Benecke R, Meyer BU, Dressler D (2003). Brain parenchyma sonography discriminates Parkinson’s disease and atypical parkinsonian syndromes. Neurology.

[CR7] Walter U, Dressler D, Wolters A, Probst T, Grossman A, Benecke R (2004). Sonographic discrimination of corticobasal degeneration vs progressive supranuclear palsy. Neurology.

[CR8] Walter U, Behnke S, Eyding J, Niehaus L, Postert T, Seidel D (2007). Transcranial brain parenchyma sonography in movement disorders: state of the art. Ultrasound Med Biol.

[CR9] Wypijewska A, Gałązka-Friedman J, Bauminger ER, Wszołek ZK, Schweitzer KJ, Dickson DW (2010). Iron and reactive oxygen species activity in parkinsonian substantia nigra. Parkinsonism Relat Disord.

[CR10] Zecca L, Berg D, Arzberger T, Ruprecht P, Rausch WD, Musicco M (2005). In vivo detection of iron and neuromelanin by transcranial sonography: a new approach for early detection of substantia nigra damage. Mov Disord.

